# Supplementary material for: Factors associated with HIV testing among pregnant women in Rwanda: A nationwide cross-sectional survey
Source: PLOS Glob Public Health. 2024 Jan 18;4(1):e0002728. doi: 10.1371/journal.pgph.0002728 (PMC10795989; doi:10.1371/journal.pgph.0002728)
Supplement: Attachment — Submitted filename: Response to Reviewer comments_HIV testing 2023.pdf [file pgph.0002728.s004.pdf]

Response to Reviewer comments

Manuscript title: Factors associated with HIV testing among pregnant women in Rwanda: a nationwide cross-sectional survey

**Reviewer comment**	**Response**	**Line**
Thank you for submitting your manuscript to PLOS Global Public Health. After careful consideration, one of the reviewers feels that it has merit but does not fully meet PLOS Global Public Health's publication criteria as it currently stands. Therefore, we invite you to submit a revised version of the manuscript that addresses the points raised during the review process. Consider the reviewer's comments on how to improve the sections on sampling and your model testing. Also ensure that there are no typos left such as "Reproductive age" (P.4, Line 89) with "reproductive" that should not be capitalised, or the punctuation in "The included;" (P.7, Line 147), there should not be a semicolon. Review thoroughly your sentences.	Thank you for this second round of peer review. We have carefully considered reviewer's comments and suggestions in the revised version. Appropriate revisions have been made and details provided regarding sampling and model testing for clarity.<br>We have also revised the entire paper for typos and grammar mistakes as suggested.	
Reviewer #2: All comments have been addressed<br>Reviewer #2: This article has greatly improved from its last version and I would recommend its publication. It is more understandable to readers/public. If statistician/epidemiologist agrees with the presented statistical findings	Thank you for the constructive feedback and time take to review our paper.	
**Reviewer #3: Overall Impression:**<br>The article on the prevalence and factors associated with HIV testing among pregnant women in Rwanda provides valuable insights. The study is commendable for its comprehensive analysis and	Thank you for the positive feed back and time taken to review our manuscript.<br>Your suggestions and comments have been considered to improve the clarity and rigor of our study.	

use of recent data from the 2020 Rwanda Demographic and Health Survey. The abstract and results sections are well-structured and present clear findings. However, there are several areas that could benefit from improvement to enhance the clarity and rigor of the study		
**Areas for Improvement:**<br>- The article displays commendable strengths, utilizing recent data and employing a robust sample size with standardized protocols. However, clarity is needed on the entity conducting multistage stratified sampling.<br><br>- The abstract could benefit from refined language.<br><br>-Questions arise regarding model testing methodology and variable selection criteria.	We have clarified the entity in the abstract as suggested "Multistage stratified sampling was used by RDHS team to select participants."<br><br>We have also refined the language used in abstract specifying what RDHS did and what researchers did.<br><br>We have specified how model testing was done "Model fitness was assessed with Hosmer-Let me show test, where small Chi-squared values (with larger p-value closer to 1) implied significant model fitness"<br>We have also explained how variable selection was done for multivariable logistic regression, giving justification for the approach; "Independent variables found significant at a p-value <0.25 were then included in the multivariable model, including those reported to be significantly associated with HIV testing in previous studies, regardless of their significance on bivariable analysis. In other	

	words, all variables in bivariable analysis were included in multivariable logistic regression model. This model approach was used to avoid possible elimination of factors which although may be non-significant on bivariable analysis but may become significant on adjusting for other variables in the model.”	
-Attention is required to accurate referencing and contextual integration, avoiding over-generalizations.	Thank you for this suggestion. We have revised our references for accurate referencing using primary studies. In addition, we have revised the manuscript to make sure that results are explained and conclusions drawn from results and related to the context of the paper and specific study groups under study.	
-Structural improvements, such as breaking down lengthy paragraphs and contextual integration, can enhance readability.	These have been considered in the revised draft as suggested. In addition, countries where the literature is obtained and the specific groups of the study are also highlighted.	
-Ethical considerations, especially the need for institutional approval, require clarification.	We have revised the ethical considerations clarifying that additional approval was waived by DHS due to the use of secondary data in this study.	
-Variable definitions, like "parity," should be clearer, and consistency in presenting confidence intervals is essential.	We have revised parity to a clearer term “number of children ever born”. We have also	

-The discussion section would benefit from improved sentence structure.<br><br>-Addressing contradictory findings and emphasizing recall bias impact can strengthen the article.<br><br>-A more extensive discussion on study limitations, particularly the cross-sectional design, would enhance the overall quality of the manuscript.	revised the presentation of confidence intervals for consistence as suggested.<br><br>We have revised the discussion with improved sentence structure, as suggested.<br><br>Contradictory findings have been addressed and we have included the impact of potential recall bias in the limitations section. “the risk of missing information and recall bias due to the sensitivity of the HIV topic (prone to social desirability effects) and data was self-reported, without verifying with health records. All this could have affected the true estimation of HIV testing prevalence.”<br><br>As suggested, we have revised the limitation section to include details of cross-sectional design as a limitation, and its implication.	
**Specific comments:**<br>- Clarify whether multistage stratified sampling was conducted by researchers or the RDHS team unaffiliated with the study, specifying the role of the USAID RDHS team.	We have clarified who performed stratified sampling as suggested “Multistage stratified sampling was used by RDHS team to select participants.”<br>USAID RDHS team was responsible for the whole aspects of the survey including IRB submissions, selection of study participants, and data collection. The authors of the present study conducted a secondary analysis of already available RDHS dataset.	

- Enhance precision in the abstract by specifying if univariable models were tested with all explanatory variables.	We have included this as suggested; "We conducted bivariable and multivariable logistic regression to explore factors associated with HIV testing"	
- Provide details on model testing, including whether AIC or BIC was used and the criteria for selecting factors in the model.	We have specified how model testing was done "Model fitness was assessed with Hosmer-Lemeshow test, where small Chi-squared values (with larger p-value closer to 1) implied significant model fitness". The suggested AIC and BIC are more applicable to structural equation modelling, and these were not used in this case.<br>We have also explained how variable selection was done for multivariable logistic regression, giving justification for the approach; "Independent variables found significant at a p-value <0.25 were then included in the multivariable model, including those reported to be significantly associated with HIV testing in previous studies, regardless of their significance on bivariable analysis. In other words, all variables in bivariable analysis were included in multivariable logistic regression model. This model approach was used to avoid possible elimination of factors which although may be non-significant on bivariable analysis but may become significant on adjusting for other variables in the model. Backward elimination was then executed until the model	

	contained only significant variables with p-value <0.05." [1].<br>[1]. Bursac Z, Gauss CH, Williams DK, Hosmer DW. Purposeful selection of variables in logistic regression. Source code for biology and medicine. 2008 Dec;3(1):1-8.	
- Discuss the limitations of using odds ratio in a cross-sectional study. Usually, it is impossible to use a odds ratio in a cross sectional study and you use a prevalence of odds ratio.	Thank you for this insightful suggestion. We have briefly discussed the limitation of using odds ratios in cross-sectional studies in the limitations section. "the interpretation of odds ratios in this study should be done with caution given the cross-sectional nature of the study, so they should be framed in terms of odds, not in terms of probabilities since they don't imply relative risk."	
- Verify reference accuracy, check reference 8. Because you say in the sentence "In most African countries…", however the study you are referencing is just in Zimbabwe. Make sure you are referencing the actual study and not a study within a study. Because otherwise this is a very huge generalization.	After careful consideration, we eliminated the sentence	
- Improve the structure of the introduction by separating the first paragraph into more manageable sections.	The first paragraph has been broken down into smaller paragraphs, as suggested.	
- Review references, particularly in instances like reference 16, ensuring accurate citation of the original studies and reports. You say in	Thank you for the keen observation. We have updated the content and the citation as advised.	60-63

Line 76 “Globally,….” And then reference 16, the study in reference 16 is just situated in East Africa. Be careful and review your citations and make sure to cite the original article or report.		
- Integrate statistics more effectively into the text, particularly in the paragraph starting on line 85. Like for example, “Due to the high HIV rates in Rwanda, the government of Rwanda tried to promote different initiatives which include the following x, y and z”. The statistics that you present to help paint a picture are needed but they need to be better integrated into the text.	Thank you for your concern. The paragraph containing this sentence has been deleted because it was reporting HIV statistics among the general population and not among women during pregnancy.	
- Consider starting a new paragraph for the section beginning on line 98.	A new paragraph has been started as suggested.	
- Clarify the criteria for including only pregnant women in the survey, addressing any limitations, and explaining the exclusion of women who recently gave birth. How was it determined that only pregnant women interviewed during the survey were included? Is there a particular question in the questionnaire used? Could this be a limitation? Why not include women who recently given birth?	During the RDHS women were asked “Are you currently pregnant?”, and this enabled us to filter out only pregnant women at the time of the survey. Given the study scope, we decided to exclude women who had recently given birth to ensure that our findings and recommendations are specific to pregnant women.<br>Considering that the main message of the study is to promote HIV testing during pregnancy not after pregnancy when consequences may	

	be irreversible. Therefore, we believe using only pregnant women in our analysis was not a limitation.	
- Place the ethical considerations section at the end of the methods section and justify any lack of institutional ethics approval for secondary data analysis. This might be a flaw in the ethics but even though the data is available by the DHS, usually there still needs to be a ethics approval from your institution for doing a secondary data analysis? It doesn't usually require a full review by the board but it still needs to be completed. Justification may be needed as to why this was not sought out?	As suggested, ethics consideration section has been transferred to the end of the methods section. We have also justified why additional institutional approval was not required. "Since this was a secondary data analysis of a publicly available dataset, additional ethical approval was waived by RDHS. The RDHS data set was obtained from the MEASURE DHS website (URL: https://www.dhsprogram.com/data/available-datasets.cfm) after getting their written permission.<br>The same approach has been used in all the DHS related studies published online, so we believe its not a flaw.	
- Provide information on whether researchers in the project are affiliated with the DHS team for this survey.	As suggested, this as been specified at the end of ethical considerations section. "Researchers of this project are not affiliated with the DHS team for this survey."	
- Include a citation for the Hosmer-Lemeshow test in the relevant section.	This has been included as suggested.<br>Nattino G, Pennell ML, Lemeshow S. Assessing the goodness of fit of logistic regression models in large samples: A modification of the Hosmer-Lemeshow test. Biometrics. 2020 Jun;76(2):549-60.	

- Define the health insurance variable and clarify whether it includes both public and private insurance.	This has been defined as suggested. "Health insurance was considered if a respondent had any type of insurance that covers the whole or a part of the risk incurred from medical expenses, and included both public and private insurance."	
- Consider using a term other than "parity" for better audience understanding.	We have revised parity to a clearer term "number of children ever born".	
- Reframe the distance to a health facility variable to more accurately represent the question posed, such as "Perceived problems of distance from the household to the nearby health facility."	Thank you for this suggestion, and we have reframed as suggested	
- Evaluate the placement of confidence intervals in Table 1, considering whether it might be more appropriate in the results description. Why was the confidence interval only listed for the variable "ever tested for HIV"? Seems a bit misplaced. I would say this statistic in the results description rather than the table.	Thank you for this suggestion. For clarity, we have removed the confidence interval of ever tested for HIV from table 1. We had included its confidence interval because it is our outcome variable, but since it may cause confusion, we have restricted it to results section as suggested.	
- It is unclear which variables were included in the multivariable model. Were all the variables that had crude odds included? No variables were excluded from the multivariable model? Please justify and say more. Wouldn't parity, health insurance,	We have also clarified how variable selection was done for multivariable logistic regression, giving justification for the approach. "Independent variables found significant at a p-value <0.25 were then included in the	

wealth index, residence, sex of household head, husband's education, exposure to media, distance to a health facility, STI history, healthcare decision making, comprehensive MTCT knowledge, HIV related stigma, know about HIV tests not be included in the multivariable model since they were not found to be significant with the crude bivariable associations?	multivariable model, including those reported to be significantly associated with HIV testing in previous studies, regardless of their significance on bivariable analysis. In other words, all variables in bivariable analysis were included in multivariable logistic regression model. This model approach was used to avoid possible elimination of factors which although may be non-significant on bivariable analysis but may become significant on adjusting for other variables in the model. Backward elimination was then executed until the model contained only significant variables with p-value <0.05." [1].<br>[1]. Bursac Z, Gauss CH, Williams DK, Hosmer DW. Purposeful selection of variables in logistic regression. Source code for biology and medicine. 2008 Dec;3(1):1-8.	
- Please verify model testing with AIC/BIC. There seems to still be issues with your statistical modelling.	We have specified how model testing was done "Model fitness was assessed with Hosmer-Let me show test, where small Chi-squared values (with larger p-value closer to 1) implied significant model fitness". The suggested AIC and BIC are more applicable to structural equation modelling, so were not used in this case.	
- Ensure consistency in presenting spaces within confidence intervals.<br>-	Thank you for this keen observation. We have revised the presentation of results for	

- Add "(years old)" next to the age variable for clarity.<br><br>- Improve sentence structure in the discussion section, address contradictory findings, and speculate on potential reasons for discrepancies.<br><br>- Emphasize the potential impact of recall bias on self-reported data in the limitations section.<br><br>- Expand the discussion of the cross-sectional design's limitations, considering implications for drawing causal inferences.	consistence in spaces within confidence intervals.<br><br>This has been added as suggested.<br><br>We have tried to improve the structure of some sentences in the discussion. Contradictory findings were addressed as well.<br><br>This has been emphasized as suggested. “the risk of missing information and recall bias due to the sensitivity of the HIV topic (prone to social desirability effects) and data was self-reported, without verifying with health records. All this could have affected the true estimation of HIV testing prevalence.”<br><br>We have expanded the limitation of the cross sectional design as suggested. “The cross-sectional design of this study also limits inferring causality but rather only association. Therefore, the interpretation of odds ratios in this study should be done with caution given the cross-sectional nature of the study, so they should be framed in terms of odds, not in terms of probabilities since they don’t imply relative risk.”	
