# Supplementary material for: Factors associated with HIV testing among pregnant women in Rwanda: A nationwide cross-sectional survey
Source: PLOS Glob Public Health. 2024 Jan 18;4(1):e0002728. doi: 10.1371/journal.pgph.0002728 (PMC10795989; doi:10.1371/journal.pgph.0002728)
Supplement: Attachment — Submitted filename: Response to Reviewers.docx [file pgph.0002728.s002.docx]

**Response to Reviewer comments**

**Manuscript title: Human Immunodeficiency Virus testing and associated factors among pregnant women in Rwanda:** **a nationwide cross-sectional survey**

| **Reviewer comment** | **Response** | **Line** |
| --- | --- | --- |
| **Reviewer #1: Summary of the research and overall impression:** First, I want to thank the authors for this article that hopes to address some important gaps in understanding the factors related to HIV testing in pregnant women in Rwanda. The use of data from the Rwanda Demographic and Health Survey for this secondary analysis provides valuable insights into the frequency and associations of factors related to HIV testing. However, after a careful review of the article, I believe that it requires a major revision. The writing style and presentation of the paper need improvement to enhance its clarity and coherence. Additionally, the statistical soundness of the paper should be carefully addressed to ensure the validity of the findings and the robustness of the conclusions. | Thank you very much for taking time to review our article.  The suggested revisions have been done to improve the quality of the paper. The writing style and presentation of the paper were also improved following your valuable comments to enhance its clarity and coherence.  Additionally, the suggested statistical revisions have been done to ensure the validity of the findings and the robustness of the conclusions. |  |
| Discussion of specific areas for improvement - Clarity and Writing Style: The article would benefit from a more concise and clear writing style. Complex sentences and overly technical language may hinder the readers' comprehension of the study's objectives and findings. Simplifying the language and providing clearer explanations of statistical methods would enhance the paper's accessibility. | Thank you for this suggestion. In the revised manuscript, we have revised the writing style and simplified complex sentences for clarity, as suggested. |  |
| - Statistical Analysis: While the paper presents associations between various factors and HIV testing, the statistical methodologies employed need to be rigorously reviewed. Please ensure that appropriate statistical tests and adjustments have been applied to support the validity of the results. | We have revised the statistical methodology of the paper following your suggestions. Additional analysis for the distribution of the outcome variable among sociodemographics was also included in the revised draft as suggested. Appropriate adjustments were considered in the multivariable logistic regression. | 169-237 |
| - Discussion of Findings: The paper should offer a more in-depth discussion of the findings, comparing them to existing literature and discussing their implications for public health in Rwanda. Emphasizing the unique contributions of this study will enrich the discussion section. | We have revised the discussion to provide a more in-depth evaluation of the findings. The implication of study findings for public health and the study’s unique contribution were also included in the revised draft as suggested. | 240-365 |
| - Suggestions for Future Research: Consider including suggestions for future research to address any remaining gaps or explore related aspects of HIV testing among pregnant women. Proposing areas for further investigation will add value to the article. | Thank you for this valuable suggestion. We have included some suggestions for future research in the limitation section of the revised draft. “ Moreover, the secondary data used in this study couldn't fully explore all potential determinants of HIV testing in pregnancy and couldn’t explain underlying reasons. We therefore recommend future qualitative studies to fully examine the underlying reasons for failure to take HIV testing among pregnant women in the context of Rwanda.” | 339-346 |
| More Specific Comments: - Consider revising the title to use the acronym for HIV, which would make it more concise and familiar to readers. | The title was revised and rephrased as suggested; “Factors associated with HIV testing among pregnant women in Rwanda: a nationwide cross-sectional survey”. | 1-2 |
| - The abstract should be improved to enhance readability and clarity. The excessive use of AORs makes it challenging to grasp the main results. Consider rephrasing to present the key findings more succinctly and clearly. | Thank you for this suggestion. The abstract was improved and AORs were deleted. It was paraphrased as below.  “Of the 870 pregnant women, 94.0% had tested for HIV during their current pregnancy. Younger age 24-34 years, not working, large household size, multiple sex partners, as well as secondary, primary and no education were associated with higher odds of HIV testing compared to their respective counterparts. However, being unmarried, belonging to the western region, having not visited a health facility, and not having comprehensive HIV knowledge were associated with lower odds of HIV testing.” | 36-41 |
| - Abstract conclusion: Provide more context in the conclusion about the implications of the associations and factors found in the study. Discuss their potential significance and relevance to public health and policy in Rwanda. | The statement was revised as suggested: “The study revealed that individual level factors had the greatest influence on HIV testing in pregnancy with a few household-level factors showing significance. There is need for maternal health stakeholders to design and develop HIV testing programs and policies that are regional sensitive, and these should target older, more educated, working and unmarried women with limited HIV knowledge.” | 42-46 |
| - Introduction, line 52-53: Clarify why women yield the largest burden of HIV in line 52-53. Provide a contextual explanation of the factors contributing to the higher prevalence of HIV in women in sub-Saharan Africa. Also, verify the appropriateness of reference 2. | Thank you for this comment. The contextual reasons why women yield the highest burden of HIV were incorporated in the revised draft as below; “Several factors increase women’s susceptibility to HIV acquisition including biological, behavioural, social economic, cultural and structural risks [7]. In most African countries, there are high rates of sexism where men dominate in culture and society and this aggravates women’s inferiority and in most cases women don’t play a role in sexual decision making [8]. Besides, demeaning cultural practices for example virginity tests which consequently make young women to indulge in risky anal sexual behaviours to avoid failing the test [9], poverty, gender-based violence and inequality collectively pose a high risk for HIV infection among African women [10, 11] | 59-66 |
| - Introduction, first paragraph: Explicitly state why pregnant women matter regarding HIV testing. Highlight the significance of understanding HIV testing among this specific group. | Thank you for this suggestion. The reasons why pregnant women matter regarding HIV testing were incorporated in the statement as; “HIV testing during pregnancy is crucial in preventing child maternal transmission of HIV, as it enables timely detection of HIV, rapid initiation of treatment and support during and after childbirth” | 55-57 |
| - Appropriateness of references: Verify the appropriateness of references used in the introduction. For example, check if reference 7 is suitable and consider using references from sub-Saharan Africa for more relevance. | Reference 7 was deducted and we considered references (5,6,7) | 57-61 |
| - Line 63: should be integrated into the first paragraph since it is about presenting the statistics | The statement was integrated in the first paragraph as below; “In 2021 alone, 54% of the 38.4 million people living with HIV (PLWH) worldwide in 2021 were women and girls, pregnant women inclusive and 15% of these were not aware of their HIV status “. | 51-53 |
| - Sentence 65: Reframe line 65 to avoid presenting a global statistic and then moving to sub-Saharan Africa in the same sentence. Clearly present the percentage or prevalence of children who get transmitted HIV from their HIV-positive mothers. | Thank you for this suggestion. We have revised the paragraph as suggested:  “Globally, less than 90% of pregnant women are tested for HIV [21]. In sub-Saharan Africa, over 180,000 children are infected with HIV during pregnancy, childbirth and breastfeeding by mothers aged 15-49 [16, 17, 22].” | 76-78 |
| - Line 67: Review and improve sentence structures and paragraph organization for better flow and coherence. | We appreciate your guidance. The statement was rephrased as; The later reports a great risk of MTCT of HIV during pregnancy ranging from 15-45% without antiretroviral therapy (ART), but this can be reduced to less than 5% with prenatal testing of pregnant women for HIV and early linkage to HIV care [23-25]. | 78-80 |
| - Line 74: Third paragraph needs work. Provide context for the studies referenced in line 74. Explain the relevance of these studies and their findings to the current research. | The statement was rephrased as; “A study in Rwanda among adolescent girls and young women and another study in sub-Saharan Africa among women…..” | 66-71 |
| - Line 79: Specify when Rwanda developed strategies to promote HIV testing services among pregnant women and the reasons behind their implementation. | The statement was rephrased as; “The government of Rwanda through the ministry of health (MoH) adopted strategies to promote HIV testing services among through the National Strategic Plan (NSP) of July 2013-June 2018 including mandatory HIV testing during ANC, community-based testing, self-testing and door-to-door testing [8, 7]. NSP aimed at zero new HIV infection, zero HIV related deaths and zero stigma and discrimination due to HIV (RBC 2013).” | 90-94 |
| - Line 82: Clarify line 82 by specifying whether it refers to all components of HIV testing or all components of ANC. | The statement refers to HIV testing as a component of ANC. Therefore, the statement was rephrased as; “Although HIV testing remains one of the commonest components of ANC, only 13.1% of women in Rwanda receive all the components of ANC”. | 94-95 |
| - Line 83: just need to present one statistic, the four visits since that is the common indicator of good common amount of ANC visits | We deleted the statistics of the 8 ANC visits, and rephrased the statement as below; “Indeed, 47.2% of pregnant women in Rwanda utilize at least four ANC contacts, which limits the opportunities for access to HIV testing services by pregnant women”. | 96-98 |
| - Line 85: This statement is wrong because of the articles you referenced actually does look at pregnancy as a factor. <https://bmcpublichealth.biomedcentral.com/articles/10.1186/s12889-022-13679-8> | Thanks for the great observation. We reviewed the articles and rephrased the statement to; “Even though several previous studies in Rwanda have looked at HIV testing among the general population and other groups [8, 22], “limited accessible studies have focused on pregnant women so far”. | 98-101 |
| - Introduction: I would like to inquire about previous statistics on HIV testing in Rwanda in general, not just among pregnant women. Additionally, it would be helpful to understand the reasons for focusing solely on women in this study. Could you provide statistics on HIV testing rates for women compared to men in Rwanda to provide better context for the rationale behind studying this specific population group? More contextual information on HIV testing trends among different demographics in Rwanda would strengthen the justification for the focus on pregnant women in this research. | Thank you for this comment.  Specific statistics on HIV testing among different groups (i.e. man and women) can hardly be sourced. However, we agree with the reviewer and have included more details to strengthen the rationale of focusing on pregnant women in the revised draft.” |  |
| - Line 90: don’t need a hyphen in “most-recent” | The hyphen was deleted and we paraphrased as follows;  “Therefore, this study aimed to determine the prevalence and factors associated with HIV testing among pregnant women in Rwanda, using the most recent 2020 Rwanda Demographic and Health survey.” | 106-108 |
| - Line 95: stick to one form of presentation of the objectives, you presented it in the paragraph above, don’t need to present it again below | Thank you for your guidance. This paragraph was representing objectives again and we have deleted from line 95-99 |  |
| - Introduction or method: The introduction or method section would benefit from including more contextual factors that could impact HIV testing in Rwanda. Providing information about the population size of Rwanda and the incidence of HIV in the country would help readers understand the broader context of the study. Additionally, discussing factors, both structural and social, that have been known to influence HIV testing rates in Rwanda would add valuable insights to the research. This contextual information is essential for a comprehensive understanding of the factors influencing HIV testing behavior in the specific setting of Rwanda | Thank you for this comment. As suggested, we have included contextual factors known to influence HIV testing among pregnant women from previous studies.  Information about population size and incidence of HIV have been included in the revised draft. | 101-105  85-89 |
| - Line 119: Check your references. I don’t think reference 23 is meant to be here? | Thank you. Reference 23 which is now 36 is for the RDHS, a citation for the definition and recoding of the outcome variable (HIV testing) | 147 |
| - Methodological errors should be addressed in the study. Given that the variables are described as community, household, and individual, a multilevel analysis should be considered to account for potential statistical confounding within these levels. | Thank you for this insightful suggestion. Although our study considered factors at different levels, multilevel regression analysis was not opted for because of its several limitations such as raising issues on the conceptual distinction among different levels of variables, which in real world all levels are interconnected without distinctions. Nonetheless, we used complex samples package that accounted for the DHS stratification and multi-stage sampling design there by accounting for potential statistical confounding within these levels. Our decision was guided by this methodology paper:  [1]. Diez-Roux AV. Multilevel analysis in public health research. Annual review of public health. 2000 May;21(1):171-92. | 170-190 |
| - Methods: What was the ethics procedure to obtain this data? Was there any procedures in place to do a secondary data analysis? Describe the ethical procedures for obtaining the data and address any ethical considerations in conducting a secondary data analysis. | Thank you. Ethical considerations and procedures to obtain this data have been included in the methods section of the revised manuscript. | 128-137 |
| - Additionally, missing data is common in DHS data. It would be valuable to provide information on whether any data was removed due to missing values and detail the procedure used for handling missing data. Transparent reporting on the treatment of missing data is essential to ensure the robustness and reliability of the study's results. | Thank you this suggestion. We have added details of how missing data was dealt with in the methods section. “Missing data in explanatory variables were handled by a list-wise deletion in SPSS”. | 190 |
| - Table 1: Regarding Table 1, there are some discrepancies in the numbers presented. For instance, the total number of women in the "age" category is listed as 869, which seems inconsistent with the sample size mentioned elsewhere in the article. To enhance clarity, it would be helpful to include "(years old)" next to the age variable for better understanding. | We are sorry for this slight mistake. We have carefully revised Table 1 and corrected the inconsistency. “(years old)” has also been added to the age category as suggested. | 205 |
| Additionally, the education level should ideally be presented in the order of no education first, followed by primary, secondary, and tertiary. Moreover, the representation of missing values in the table should be specified for each sociodemographic variable to provide a comprehensive overview of missing data. | The arrangement of education level subcategories have been revised as suggested in Table 1. Husband’s education and Healthcare decision making both had 134 missing values, that is why both were denoted by “a”. However, we have represented them with different denotions as suggested. | 205-207 |
| Another point of concern is the identical number of missing values for both "healthcare decision making" and "husband education." It would be beneficial to clarify why these variables have the same amount of missing data, as this could potentially indicate issues with data quality or collection. | The identical number of missing values has been clarified in the Table footnotes “Husband’s education and Healthcare decision making were both collected by the domestic violence questionnaire thus have same number of missing values” | 206-207 |
| - Table 2: There are several issues with Table 2 that need to be addressed. Firstly, if bold formatting is used to distinguish between significant and non-significant results for adjusted analyses, the same formatting should be applied to the unadjusted analyses for consistency. | Thank you. For clarity, we have revised the definition of bold in the Table foot notes to highlight only significant adjusted factors. “Bold= significant adjusted factors” | 235 |
| Furthermore, the table lacks the presentation of frequencies of sociodemographic variables across the outcome variable, which is crucial for understanding the distribution and characteristics of the study population. | Thank you for this suggestion. We have included the distribution of HIV testing across sociodemographic variables in Table 2. | 234-235 |
| Additionally, the rationale behind including all variables in the multivariable model needs clarification. It is essential to provide a clear explanation of the model building procedure and the statistical justification for including specific variables. Without this information, the soundness and validity of the multivariable model may be questioned. | Thank you for this insightful suggestion. A basic logistic regression model was used which included all selected factors (ie. all significant variables on bivariable analysis and those known to be potential predictors of HIV testing from previous studies-regardless of their significance on bivariable analysis). The justification for this model has been included in the methods section as suggested. “This model approach was used to avoid possible elimination of factors which may although be non-significant on bivariable analysis but may become significant on adjusting for other variables in the model.” | 183-185 |
| - Discussion: In the discussion, provide a summary of the data's significance in the context of existing knowledge. Discuss why this data matters and suggest potential future research directions based on the study's findings. | Thank you for this valuable suggestion. We have revised the discussion to provide a summary of the study’s significance in the context of existing knowledge.  Potential future research directions/ recommendations have also been included in the revised draft. | 239-365 |
| - Limitations line 292: sentence looks like it is missing something. It is no clear what you want to mean? | The statement was missing the word “missing” so it was rephrased as; “ However, this study also had some limitations such as the risk of missing information and recall bias due to the sensitivity of the HIV topic (prone to social desirability effects) and data was self-reported, without verifying with health records”. | 334-337 |
| - Limitations: Discuss potential limitations with the model used in the study and address any potential sources of bias. | Thank you. Potential imitations of the model and sources of bias were included in the revised draft:  -“Moreover the logistic regression model used in our analysis assumes a linear relationship between the predictor and the outcome variable, which may not be the case in real settings. Some of the predictors of HIV testing may be mediated by other factors, thus future studies on the topic should consider using more rigorous analytical methods such as mediation analysis and structural equation modeling.”  -“However, this study also had some limitations such as the risk of missing information and recall bias due to the sensitivity of the HIV topic (prone to social desirability effects) and data was self-reported, without verifying with health records. All this could have affected the true estimation of HIV testing prevalence.” | 342-346  334-338 |
| - Clarify how the results of this study will advance research on the topic and suggest next steps or potential follow-up analyses based on the study's outcomes. | Thank you for this suggestion. We have clarified how this study could advance research on the topic by including recommendations for future research directions. | 340-346 |
| **Reviewer #2: Some comments** 1. I would suggest to reframe the title by emphasizing “Factors associated to HIV testing….”. This adjustment will make your title more concise and clearer. I’m suggesting this based on your study description, results and discussion. | The title as been rephrased as; “Factors associated with HIV testing among pregnant women in Rwanda: a nationwide cross-sectional survey”. | 1-2 |
| 2. Pages 3-4, lines 51-88: I would suggest you reorganize the background. Start with global, LMICs & Africa, sub-Saharan Africa and Rwanda vis-a vis the existing knowledge about HIV testing among pregnant women….. this would be helpful for a well-framed background. | Thank you for this suggestion. The background was reorganized to start with global, LMICs and Africa | 50-110 |
| 3. Page 4, line 86: I suggest to do not use “no accessible study” but a different wording like “a limited number of studies, a couple of studies or few accessible studies or any other appropriate wording….” In fact, Rwanda has history of some studies that covered this topic at some extent and helped to achieve good outcomes in HTC among pregnant women such as Musekiwa et al. on prevalence and factors associated with self-reported HIV testing among adolescent girls and young women; Allen et al. on pregnancy and contraception after HIV testing and counseling; Mugwaneza et al. on ART and PMTCT; Kowalczyk et al. on voluntary counseling and testing (VCT) for HIV among pregnant women; and others. | The statement as been rephrased as; Even though several previous studies in Rwanda have looked at HIV testing among the general population and other groups [8, 22], limited accessible studies have focused on pregnant women so far. | 98-100 |
| 4. Page 5, line 102: Does H in RDHS stand for Health? If so, add it. | Thank you for this observation. Yes, H stands for Health and the statement as been rephrased to add “Health” in it. | 114 |
| 5. For your data sampling and analysis; were you part of RDHS team? If not, how did you collaborate with them? It would be good to mention how you accessed RDHS data (permission, ethics, etc.,). | Thank you very much. Ethical considerations and procedure for obtaining the RDHS dataset have been included in the methods section in the revised manuscript. | 128-137 |
| 6. Page 6, lines 134-136: What was considered as comprehensive knowledge of HIV/MTCT? Unless I missed it, I didn’t see such knowledge components/items. | The definitions of comprehensive HIV and MTCT knowledge were included in the “Explanatory variables” section in methods; “Comprehensive knowledge of HIV was assessed using a six-item questionnaire: three on HIV prevention information and three questions on misconceptions of HIV transmission modes. Comprehensive knowledge of MTCT of HIV was confirmed when a woman reported knowing about the possibility of HIV transmission from HIV positive mother to her child during pregnancy, delivery, and breastfeeding (that is, scoring all three questions correctly)”. | 160-167 |
| 7. Page 8, lines 169-171: Again, specify the components/items of HIV and MTCT knowledge you were looking for. | Thank you. These were already defined and specified in the “Explanatory variables” section in methods; “Comprehensive knowledge of HIV was assessed using a six-item questionnaire: three on HIV prevention information and three questions on misconceptions of HIV transmission modes. Comprehensive knowledge of MTCT of HIV was confirmed when a woman reported knowing about the possibility of HIV transmission from HIV positive mother to her child during pregnancy, delivery, and breastfeeding (that is, scoring all three questions correctly)”. | 160-167 |
| 8. Page 14, lines 225-230: This finding should have been well clarified through direct interviews instead of secondary data. Participants talking directly with researchers would have been interesting in terms of highlighting unique and contextualized factors other than those condensed in the RDHS report. Other regions in the country are rural or remote but still perform better in HTC. | We agree with the reviewer. This has been included in the study limitations and recommendations for future research. | 332-346 |
| 9. Page 10, lines 186-188 and page 16, lines 283-285: This is a good example of how specific questions directed to participants would have unpacked or given a clear idea about different factors were associated to HTC among pregnant women. May be these findings will be a starting point for your next study using primary data! | We agree with the reviewer and thanks for insightful suggestion. We shall use these findings for our future studies using primary data. |  |
