# Supplementary material for: Factors associated with HIV testing among pregnant women in Rwanda: A nationwide cross-sectional survey
Source: PLOS Glob Public Health. 2024 Jan 18;4(1):e0002728. doi: 10.1371/journal.pgph.0002728 (PMC10795989; doi:10.1371/journal.pgph.0002728)
Supplement: Attachment — Submitted filename: ManuscriptRevisions_Part2_PLOSGlobalPublicHealth.docx [file pgph.0002728.s003.docx]

Overall Impression:

The article on the prevalence and factors associated with HIV testing among pregnant women in Rwanda provides valuable insights. The study is commendable for its comprehensive analysis and use of recent data from the 2020 Rwanda Demographic and Health Survey. The abstract and results sections are well-structured and present clear findings. However, there are several areas that could benefit from improvement to enhance the clarity and rigor of the study.

Areas for Improvement:

- The article displays commendable strengths, utilizing recent data and employing a robust sample size with standardized protocols. However, clarity is needed on the entity conducting multistage stratified sampling. The abstract could benefit from refined language. Questions arise regarding model testing methodology and variable selection criteria. Attention is required to accurate referencing and contextual integration, avoiding overgeneralizations. Structural improvements, such as breaking down lengthy paragraphs and contextual integration, can enhance readability. Ethical considerations, especially the need for institutional approval, require clarification. Variable definitions, like "parity," should be clearer, and consistency in presenting confidence intervals is essential. The discussion section would benefit from improved sentence structure. Addressing contradictory findings and emphasizing recall bias impact can strengthen the article. A more extensive discussion on study limitations, particularly the cross-sectional design, would enhance the overall quality of the manuscript.

Specific comments:

- Clarify whether multistage stratified sampling was conducted by researchers or the RDHS team unaffiliated with the study, specifying the role of the USAID RDHS team.
- Enhance precision in the abstract by specifying if univariable models were tested with all explanatory variables.
- Provide details on model testing, including whether AIC or BIC was used and the criteria for selecting factors in the model.
- Discuss the limitations of using odds ratio in a cross-sectional study. Usually, it is impossible to use a odds ratio in a cross sectional study and you use a prevalence of odds ratio.
- Verify reference accuracy, check reference 8. Because you say in the sentence “In most African countries…”, however the study you are referencing is just in Zimbabwe. Make sure you are referencing the actual study and not a study within a study. Because otherwise this is a very huge generalization.
- Improve the structure of the introduction by separating the first paragraph into more manageable sections.
- Review references, particularly in instances like reference 16, ensuring accurate citation of the original studies and reports. You say in Line 76 “Globally,….” And then reference 16, the study in reference 16 is just situated in East Africa. Be careful and review your citations and make sure to cite the original article or report.
- Integrate statistics more effectively into the text, particularly in the paragraph starting on line 85. Like for example, “Due to the high HIV rates in Rwanda, the government of Rwanda tried to promote different initiatives which include the following x, y and z”. The statistics that you present to help paint a picture are needed but they need to be better integrated into the text.
- Consider starting a new paragraph for the section beginning on line 98.
- Clarify the criteria for including only pregnant women in the survey, addressing any limitations, and explaining the exclusion of women who recently gave birth. How was it determined that only pregnant women interviewed during the survey were included. Is there a particular question in the questionnaire used? Could this be a limitation? Why not include women who recently given birth?
- Place the ethical considerations section at the end of the methods section and justify any lack of institutional ethics approval for secondary data analysis. This might be a flaw in the ethics but even though the data is available by the DHS, usually there still needs to be a ethics approval from your institution for doing a secondary data analysis? It doesn’t usually require a full review by the board but it still needs to be completed. Justification may be needed as to why this was not sought out?
- Provide information on whether researchers in the project are affiliated with the DHS team for this survey.
- Include a citation for the Hosmer-Lemeshow test in the relevant section.
- Define the health insurance variable and clarify whether it includes both public and private insurance.
- Consider using a term other than "parity" for better audience understanding.
- Reframe the distance to a health facility variable to more accurately represent the question posed, such as "Perceived problems of distance from the household to the nearby health facility."
- Evaluate the placement of confidence intervals in Table 1, considering whether it might be more appropriate in the results description. Why was the confidence interval only listed for the variable “ever tested for HIV”. Seems a bit misplaced. I would say this statistic in the results description rather than the table.
- It is unclear which variables were included in the multivariable model. Were all the variables that had crude odds included? No variables were excluded form the multivariable model? Please justify and say more. Wouldn’t parity, health insurance, wealth index, residence, sex of household head, husband’s education, exposure to media, distance to a health facility, sti history, healthcare decision making, comprehensive mtct knowledge, hiv related stigma, know about hiv tests not be included in the multivariable model since they were not found to be significant with the crude bivariable associations? Please verify model testing with AIC/BIC. There seems to still be issues with your statistical modelling. Ensure consistency in presenting spaces within confidence intervals.
- Add "(years old)" next to the age variable for clarity.
- Improve sentence structure in the discussion section, address contradictory findings, and speculate on potential reasons for discrepancies.
- Emphasize the potential impact of recall bias on self-reported data in the limitations section.
- Expand the discussion of the cross-sectional design's limitations, considering implications for drawing causal inferences.
