# Supplementary material for: Factors associated with HIV testing among pregnant women in Rwanda: A nationwide cross-sectional survey
Source: PLOS Glob Public Health. 2024 Jan 18;4(1):e0002728. doi: 10.1371/journal.pgph.0002728 (PMC10795989; doi:10.1371/journal.pgph.0002728)
Supplement: Attachment — Submitted filename: Response to Editor comments NOV DEC 2023.docx [file pgph.0002728.s006.docx]

**Response to Editor comments**

**Manuscript title:** Factors associated with HIV testing among pregnant women in Rwanda: a nationwide cross-sectional survey

| **Editor comment** | **Response** | **Line/Page Number** |
| --- | --- | --- |
| Please address these two pending issues as shared with you in the previous review. Do provide references for the following passages of your third revised manuscript: Page 18, Lines 314-316; Page 18, Lines 316-318. | Thank you for your feedback.  We have added two references as advised.  49  50 | 316  318  Page 18 |
| For the following section (Page 18, Line 321 and Page 19, Lines 322-323), it is not necessarily a contradiction that both married women and women who have multiple partners have higher odds of HIV testing as married women might have more than one sexual partner and/or feel the importance of being tested for HIV, and women who have multiple partners may be single or not. Both groups of women are different, and for different reasons and contexts, they might be more likely to be tested for HIV. Please rephrase and indicate where changes were made. | Thank you for your comment.  The statement has been re-phrased as follows;  “On the other hand, due to differences in the context, studies in Ethiopia identified women with multiple sexual partners as having higher odds of HIV testing compared to those with one sexual partner, due to a higher perceived risk of acquiring HIV”. | 321-322  Pages 18 and 19 |
