# Supplementary material for: Factors associated with HIV testing among pregnant women in Rwanda: A nationwide cross-sectional survey
Source: PLOS Glob Public Health. 2024 Jan 18;4(1):e0002728. doi: 10.1371/journal.pgph.0002728 (PMC10795989; doi:10.1371/journal.pgph.0002728)
Supplement: Attachment — Submitted filename: Response to reviewer comments HIV testing November 2023.docx [file pgph.0002728.s005.docx]

**Response to Reviewer comments**

**Manuscript title:** Factors associated with HIV testing among pregnant women in Rwanda: a nationwide cross-sectional survey

| **#** | **Reviewer Comment** | **Response** | **Page /line No** |
| --- | --- | --- | --- |
| 1 | Carefully review any typos and address issues raised previously. For example, it has been asked to correct the following points in the last review, however it has not been done Page 4, Line 89, remove the capital letter in "Reproductive age", it should be written with a lower case; Page 7, Line 147: After "These included", a colon should be used, not a semicolon. | Thank you for the comment Unfortunately, we are unable to locate the comment. Kindly provide more advice on how to locate it. Thanks |  |
| 2 | Specifically, in the abstract: Line 45, instead of "regional sensitive", replace with "region-sensitive". | Thank you for the comment The word “regional sensitivity” has been changed to region-sensitive” | Pg 2  Line 45 |
| 3 | Page 3, Line 62: Stating "women's inferiority" connotes sexism. Women are not inferior to men, it is a patriachal and sexist society that marginalises women. Please rephrase your sentence. Same line: Remove the space after "inclusive". | Thank you for the comment. The word “women inferiority” has been changed to “women marginalization” | Pg 4-line 74 |
| 4 | Please remove all contractions of verbs in the text, such as "don't play" or "couldn't" and write them in full terms/verbs. | Thank you for the comment. Contractions have been removed | Pg 20 345-349 |
| 5 | Page 3, Line 64: Please change the verb "indulge" with "undergo". Rephrase this sentence, including the part with " to avoid failing the test". Women and their partners resort to other forms of sexual relationships in fear of societal discrimination and consequences? | Thank you for the comment. Changed in previous versions of the manuscript. |  |
| 6 | Page 5, Line 108: Change "special" with "specific". | Thank you for the comment. The word has been changed from "special" to "specific". | Pg 5, line 100 |
| 7 | Page 7, Line 152: after parity, you can add in parentheses "number of children" as asked by one of the reviewers. | Thank you for the comment. Parity (number of children ever born) were placed in parenthesis | Pg 6, line 135 |
| 8 | Page 17, Lines 284-286: Please add references to justify this argument. Line 285: Replace "highly" with "likely". | Thank you for the comment. Sentence was adjusted to “women are likely to have demanding careers…” | Pg 17 line 291 |
| 9 | Page 18, Lines 310-311: What are the other possible reasons, and add the references. | Thank you for the comment. Unfortunately, we are unable to locate the comments. Kindly provide more advice on how to locate it. Thanks |  |
| 10 | Page 18, Lines 322-323: The article you chose is in contradiction with the previous sentence. Please review to ensure cohehence. | Thank you for the comment. Unfortunately, we are unable to locate comment. Kindly provide more advice on how to locate it. Thanks |  |
| 11 | Page 20, Line 354: Remove the space before "interventions". | Thank you for the comment. Spaced removed | Pg 20, 363 |
